# Supplementary material for: Reference panel guided topological structure annotation of Hi-C data
Source: Nat Commun. 2022 Dec 2;13:7426. doi: 10.1038/s41467-022-35231-3 (PMC9718747; doi:10.1038/s41467-022-35231-3)
Supplement: Supplementary file 1 — Supplementary Information [file 41467_2022_35231_MOESM1_ESM.pdf]

Supplementary Information
reference panel guided topological structure annotation of Hi-C data

Supplementary Note 1. Comparison with contact map enhanced approach

Hi-C contact map enhancement has been intensively explored in recent years [19, 18, 38, 40, 4]. We can perform contact map enhancement for Hi-C contact maps and then use loop detection tools to detect loops. Here we compared RefHiC with tools that predict loops from DeepLoop [38] enhanced contact maps. DeepLoop is a deep learning approach for Hi-C contact map enhancement. It enhances or denoises loop signals from Hi-C contact maps. It outputs a list of pairs (i.e. loop clusters) with a value named LoopStrength. Although Zhang et al. [38] suggest selecting the highest LoopStrength pairs as loops, the results are still loop clusters. We called loops from DeepLoop's output by selecting loops with RefHiC's clustering method (minDelta=5 and minScore=0). Due to the absence of essential input files for running DeepLoop for contact maps aligned to hg38, we were unable to duplicate DeepLoop's result with our data (hg38), so we used DeepLoop's output (GSE167200, hg19) produced by Zhang et al. [38] for Rao's GM12878 Hi-C data. We executed RefHiC on the same data but aligned to hg38. To facilitate comparison, we lifted RefHiC's predictions over to hg19 using liftOver (https://github.com/dphansti/liftOverBedpe). Supplementary Fig. 3 shows RefHiC identified more ChIA-PET/HiCHIP-supported loop predictions than all DeepLoop-based approaches when evaluated using CTCF ChIA-PET, H3K27ac HiCHIP, and SMC1 HiCHIP data.

Supplementary Note 2. Comparison with a baseline deep learning model

Although we demonstrated that RefHiC outperformed HiCCUPS, Mustache, Chromosight, and Peakachu, these results do not imply that these gains can necessarily be attributed to the use of a reference panel. To address this question, we built a deep learning model named Baseline. The model architecture is similar to RefHiC's but without using the reference panel as input. It consists of (i) an encoder that is identical to RefHiC's and (ii) four fully connected layers with $[d,\ d/2,\ d/2,\ 1]$ units. All hidden layers are ReLU activated. Like RefHiC, we used batch normalization and dropout to regularize our model. The training data and procedure are identical to RefHiC's. We applied Baseline model to several downsampled GM12878 Hi-C contact maps and compared it with all other tools. Supplementary Fig. 4 shows that RefHiC outperformed Baseline and other tools at all sequencing depths. This result suggests RefHiC benefits from the introduction of a reference panel.

Supplementary Note 3. Additional analysis of the properties of loop predictions

To understand the specific properties of the loops predicted by each tool, we studied local interaction frequency ranks and radii of predicted loops. We found the interaction frequencies for $17-36\%$ of loops predicted by Chromosight, RefHiC, Peakachu, and Mustache were local maxima in a 3×3 region centered at loop predictions. In contrast, 75% of loops predicted by HiCCUPS were local maxima in the same region (Supplementary Fig. 6). Loops predicted by HiCCUPS had smaller radii than alternative tools (Supplementary Fig. 7). These two observations explained the diffuse loop center in Fig. 2b. Although we define loops as locally enriched significant interactions, they are not necessarily local maxima in terms of interaction frequencies. To demonstrate the validity of non-locally-optimal loops, We moved all non-locally-maximal loop predictions to locally maximal contact pairs in the surrounding 3×3 region. Supplementary Fig. 8 shows that for all comparisons excepts the HiCCUPS/RAD21, revised predictions are worse than the original predictions.

Loop size estimation

Loops form blob-shaped patterns in Hi-C contact matrices, and we can approximately represent them as circles with a minimum radius of 0.5 bin. To estimate a predicted loop's size (radius), we utilized the scale space representation [27] in computer vision by treating Hi-C contact maps around a loop prediction as an image. Briefly, for a given loop prediction at contact bins (i, j), we extracted the 21×21 Hi-C sub-matrix centred at (i, j) and used the 'blob_dog' function in scikit-image [33] to compute the radius of the blob that covers (i, j). As not all loops could be detected by 'blob_dog', we only included loops with radii found by 'blob_dog' in our analysis.

Supplementary Note 4. Applying RefHiC to novel cell types

Although we demonstrated that RefHiC does not produce more false-positive predictions than alternative tools in analyzing cohesin-depleted Hi-C data (Fig. 3a), we did not benchmark RefHiC in analyzing distant study samples. To evaluate it, we performed two different experiments (i.e. method 1, and 2). We applied both methods to detect loops from test chromosomes (chromosomes 15-17) on a downsampled GM12878 Hi-C contact (500M valid contact pairs). Method 1 uses the default human reference panel and assumes that all test chromosomes correspond to chromosome 2 in the reference panel. For instance, when computing the loop score for the pair (chr15:100000-105000, chr15:180000-185000), we extracted data for (chr2:100000-105000, chr2:180000-185000) from the reference panel. In this case, all loops in our study sample are novel. Method 2 uses a new reference panel containing eight samples. This new reference panel does not contain any samples that come from the same developmental lineage as the study sample or samples such that the similarity [37, 16] between it and the study sample is larger than 0.7. Supplementary Fig. 14 shows that RefHiC with input data configured as Method 1 performed equally well as conventional tools. At the same time, worse than RefHiC (with default reference panel) and our deep learning baseline model. RefHiC with reference panel defined as Method 2 is equally well as RefHiC (with default reference panel).

Supplementary Note 5. RefHiC outperforms a global similarity based approach

RefHiC's superior performance is achieved by its deep learning module using local information from the study sample and reference samples for topological structure annotation. It is interesting to evaluate the performance of a simple approach that uses only reference data similar to the study sample to make predictions. We first compared the study sample against each reference sample with HiCRep [37, 16] and selected 5 reference samples most similar to the study sample. We then performed Mustache and our baseline deep learning model (Supplementary Note 2) to detect loops from the five reference samples. We evaluated four different alternative approaches: Mustache top-1, predict loops as loops predicted from the most similar reference sample by Mustache, Mustache top-5, predict loops as the ensemble of loops predicted from the five reference samples by Mustache, DL-Baseline top-1, predict loops as loops predicted from the most similar reference sample by our deep learning baseline, DL-Baseline top-5, predict loops as the ensemble of loops predicted from the five reference samples by our deep learning baseline. Applying both tools to the five samples identified 19,890 (Mustache), and 32,520 (DL-Baseline) unique loops, and many of them are nearby loops within 5 bins each other (i.e. a cluster of loops) within each set. Each cluster may correspond to a single true loop. We thus used RefHiC's pooling algorithm to select a represent loop for each cluster while using the loop occurrence in the five samples as loop score. We selected 2,962 (Mustache top-5) and 3,060 (DL-Basele top-5) loops at the end (a simple voting ensemble does not work as most loops only occur once among the five samples). Supplementary Fig. 17 shows RefHiC outperformed all other approaches significantly, and some of the four proposed approaches are slightly worse than applying the corresponding tool directly to the study sample.

Supplementary Table 1. Human reference panel

ID	Sample	Valid read pairs	Source
HIC00001	22Rv1 (prostate cancer cell line)	685096962	[7]
HIC00002	293TRex-Flag-BRD4-NUT-HA	1660296754	[28]
HIC00007	BLaER (lymphoblastic leukemia cell line)	406322404	[30]
HIC00041	HCT-116 (colorectal cancer cell line)	603179292	[25]
HIC00067	HeLa Kyoto cell, MboI G1 sync control	1045885928	[35]
HIC00090	HepG2 (hepatocellular carcinoma cell line)	1759654311	[5]
HIC00091	HL60/S4 (neutrophil-like Myeloid leukemia cell line)	478434139	[11]
HIC00113	Nalm6 (B cell precursor leukemia cell line)	816274711	[32]
HIC00168	WI38_RAF (WI-38hTERT/GFP-RAF1-ER)	602556180	[29]
HIC00172	Embryonic stem cell, Cardiomyocyte differentiation : hESCs (day 0)	1914642484	[39]
HIC00183	teloHAEC (endothelial cell line)	911486437	[14]
HIC00200	Naïve human embryonic stem cells	731906045	[1]
HIC00203	GM23248 (primary skin fibroblasts)	1797370277	[23]
HIC00221	MDM (monocyte-derived macrophages)	590449106	[9]
HIC00269	Astrocytes of the cerebellum primary cell	430822244	[5]
HIC00273	HAP1 (near-haploid cell line)	413436528	[8]
HIC00280	Purified human germinal center B cells	426222299	[3]
HIC00287	Liver	447028100	[15]
HIC00295	Thymus	507309033	[15]
HIC00296	H1 Embroynic Stem Cell	989388439	[6]
HIC00310	A549 00h 100 nM dexamethasone	1548684355	[5]
HIC00318	HUVEC	438880295	[24]
HIC00319	IMR90	1053932182	[24]
HIC00320	K562	880877579	[24]
HIC00321	KBM7	877658969	[24]
HIC00322	NHEK	653628335	[24]
HIC00337	Gastric tissue	426476775	[12]
HIC00343	Left Ventricle	547477074	[12]
HIC00354	Spleen	490487515	[12]
HIC00360	GM12878	1994319522	[24]

Supplementary Table 2. Mouse reference panel

ID	Sample	Valid read pairs	Source
4DNFIKK3QG34	46C with Sox1-GFP	5370123882	[2]
4DNFIHBTUDO9	Olfactory receptor cells	2024307320	[20]
4DNFIAVHP5AV	B cell derived cell line	1869747094	[34]
4DNFIMV54HXI	Olfactory receptor cells	1585896942	[20]
4DNFI5QJNFAT	F123-CASTx129 (Tier 2)	1072601088	[13]
4DNFIS5ZK13C	CH12	1163734014	[34]
4DNFI3M6726I	Olfactory receptor cells	1202229560	[20]
4DNFIPNP9H9T	B cell derived cell line	1125298567	[34]
4DNFI3QLT3KJ	B cell derived cell line	1203634092	[34]
4DNFI6RG9TXL	ES-E14	812429482	[36]
4DNFIJLJIRKT	CH12.LX	770649212	[26]
4DNFIB8NZIAK	F123-CASTx129 with Sox2 tags and RMCE site between Sox2 and its SE	770655888	[10]
4DNFITSJBJ9G	F123-CASTx129 with Sox2 tags and RMCE site between Sox2 and its SE	759678776	[10]
4DNFIASQYF5S	CH12	915292686	[34]
4DNFI4LH8RMQ	ES-E14 with Flo/Flox deletion of Mll3 and Mll4 genes	717826154	[36]
4DNFIOPKGMBL	ES-E14	576226710	[36]
4DNFIB7RFFBB	Sertoli cell	589079877	[17]
4DNFIX524X88	ES-E14 with Flo/Flox deletion of Mll3 and Mll4 genes	693626370	[36]
4DNFI37GAU3L	ES-E14 with Flo/Flox deletion of Mll3 and Mll4 genes	579214478	[36]
4DNFIC453HVL	F123-CASTx129 (Tier 2)	514097723	[13]

Supplementary Table 3. Different datasets used in this study

Experiment	Training	Evaluation	Figure	Identifier
GM12878 CTCF ChIP-Seq		✓	Fig. 2g,h, 6b, 6f, Supplementary Fig. 10, 11, 15b, 16	ENCFF796WRU
GM12878 H3K27me3 ChIP-Seq		✓	Fig. 6g	ENCFF039JOT
GM12878 RAD21 ChIP-Seq		✓	Fig. 6f, Supplementary Fig. 16	ENCFF662DRZ
GM12878 H3K36me3 ChIP-Seq		✓	Fig. 6g	ENCFF171MDW
GM12878 SMC3 ChIP-Seq		✓	Fig. 6f, Supplementary Fig. 16	ENCFF887CRE
IMR-90 CTCF ChIA-PET		✓	Fig. 3e	ENCFF682YFU
mESC CTCF ChIP-seq		✓	Fig. 3h	ENCFF508CKL
mESC CTCF ChIA-PET		✓	Fig. 3g	ENCFF550QMW
IMR-90 CTCF ChIP-Seq		✓	Fig. 3f	ENCFF203SRF
GM12878 RAD21 ChIA-PET	✓	✓	Fig. 2e, 4d, Supplementary Fig. 4, 5, 8, 9, 12, 14, 17	ENCLB784HEF
GM12878 H3k27ac HiCHIP	✓	✓	Fig. 4f, Supplementary Fig. 2a, Supplementary Fig. 3, 4, 5, 8, 9, 12, 14, 17	[22]
GM12878 SMC1 HiCHIP	✓	✓	Fig. 2f, 4e, Supplementary Fig. 3, 4, 5, 8, 9, 12, 14, 17	[21]
GM12878 CTCF ChIA-PET	✓	✓	Fig. 2d, 4c, 6c, Supplementary Fig. 3, 4, 5, 8, 9, 12, 14, 17	[31]
K562 CTCF ChIA-PET		✓	Fig. 3b	ENCFF001THV
K562 RAD21 ChIA-PET		✓	Fig. 3c	GSM1436264
K562 CTCF ChIP-seq		✓	Fig. 3d	ENCFF119XFJ

Supplementary Table 4. TAD callers and parameters used in this study

Tool	Configuration (Parameters)
CaTCH	default (resol=5000)
EAST	EAST2, default (resol=5000)
HiTAD	default (resol=5000)
GMAP	default (resol=5000)
Armatus	-m -N -r 5000 -g 0.5 -s 0.05 -n 100
deDoc	default (resol=5000)
Grinch	-e 1000000,2000000,500000 (resol=5000)
OnTAD	-maxsz 600 (resol=5000)
Arrowhead	default (resol=5000)
DomainCall	default (resol=5000)
TopDom	default (resol=5000)
ICFinder	default (resol=5000)
HiCSeg	6 TADs per 1MB region, Gaussian distribution, block-diagonal model (resol=5000)

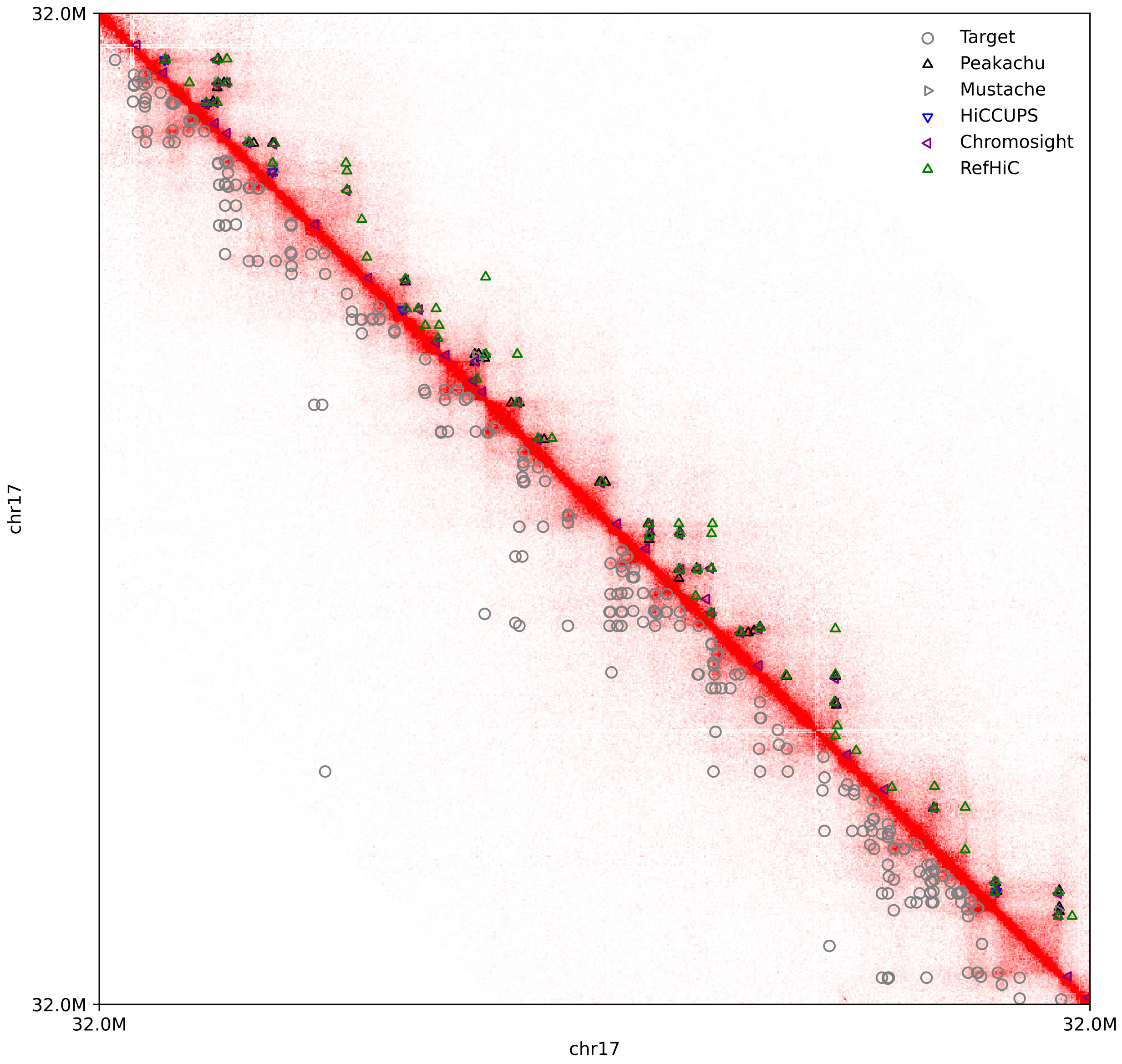

Supplementary Figure 1. Comparison of loops predicted by RefHiC, Chromosight, Peakachu, HiCCUPS, and Mustache on a small region of GM12878 HiC data (500M valid read pairs). Target annotations are loops revealed by experimental data including CTCF ChIA-PET, RAD21 ChIA-PET, SMC1 HiCHIP, and H3K27ac HiCHIP.

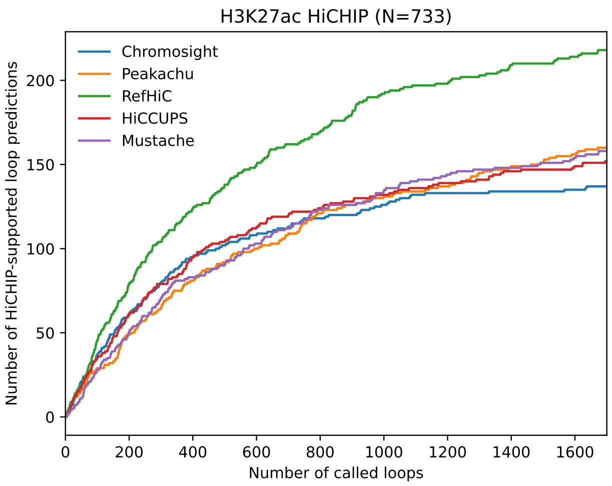

(a)

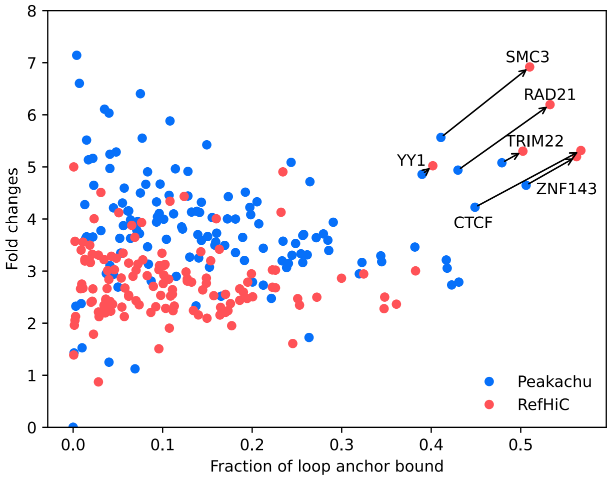

(b)

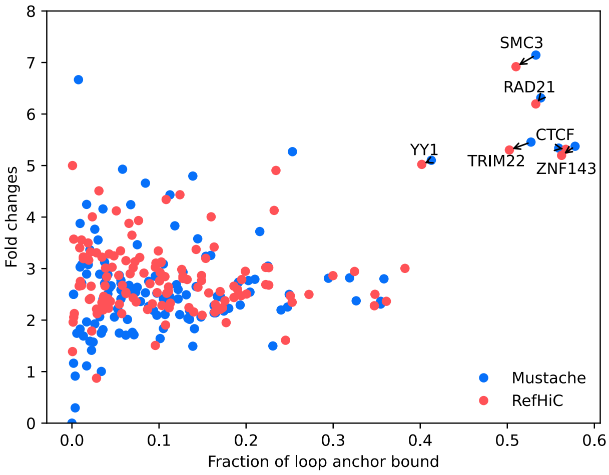

(c)

Supplementary Figure 2. Additional comparison of RefHiC, Chromosight, Peakachu, HiCCUPS, and Mustache on GM12878 HiC data (500M valid read pairs). a, Same as Fig. 2d, but compared against H3K27ac HiCHIP data. **b,** Same as Fig. 2i, but for RefHiC's and Peakachu's predictions. Though most transcription factors are more strongly enriched at Peakachu's loop predictions than at RefHiC's loop predictions, the TFs involved in loop formulations are more strongly enriched at RefHiC loop predictions. **c,** Same as c, but for RefHiC's and Mustache's prediction. The TF are enriched similarly at both types of loop predictions.

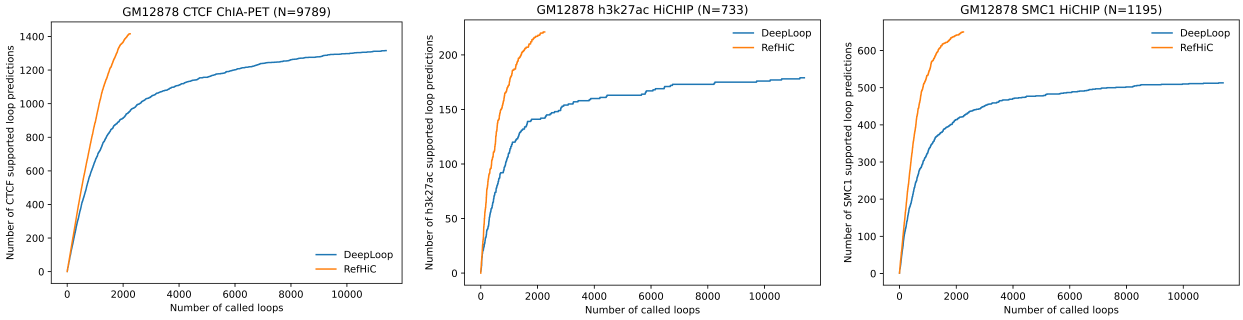

(a) (b) (c)

Supplementary Figure 3. Comparison of loops predicted by RefHiC and DeepLoop on GM12878 HiC data (2000M valid read pairs). Number of ChIA-PET/HiCHIP-supported loop predictions, among predictions made by RefHiC and DeepLoop for test chromosomes 15-17 compared against CTCF ChIA-PET (a), H3K27ac HiCHIP (b), and SMC1 HiCHIP (c). RefHiC's loop predictions matches all data better than predictions made by DeepLoop.

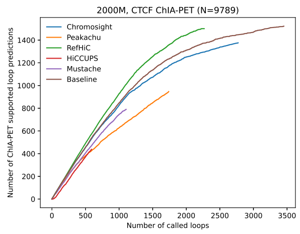

(a)

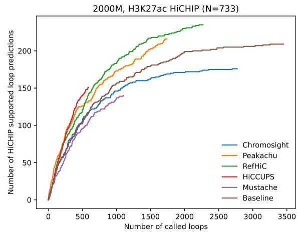

(b)

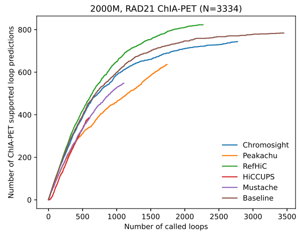

(c)

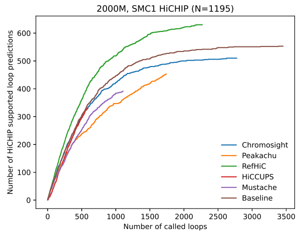

(d)

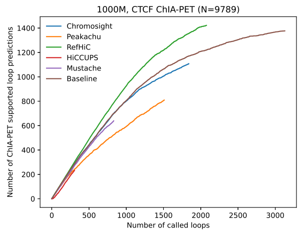

(e)

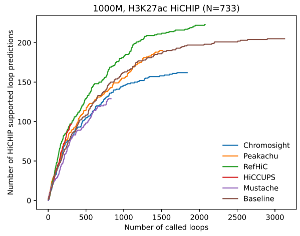

(f)

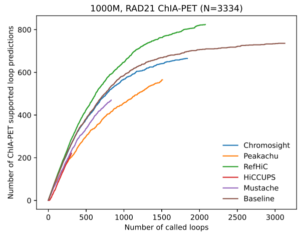

(g)

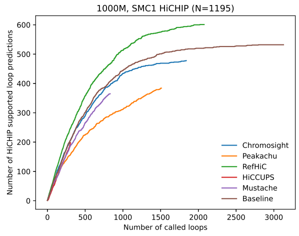

(h)

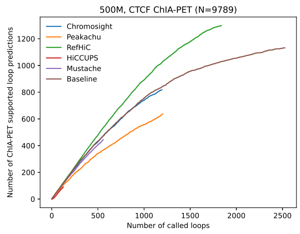

(i)

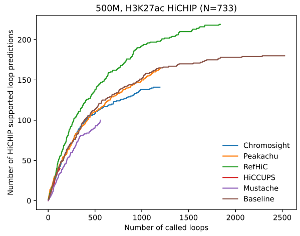

(j)

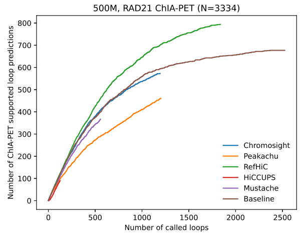

(k)

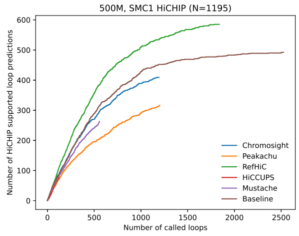

(l)

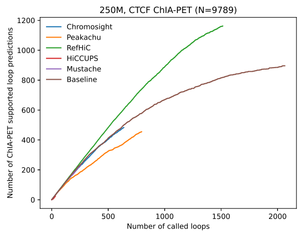

(m)

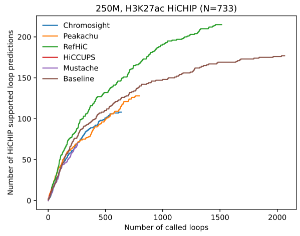

(n)

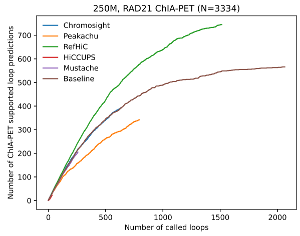

(o)

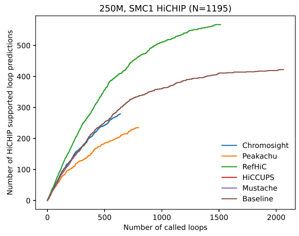

(p)

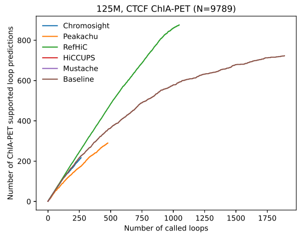

(q)

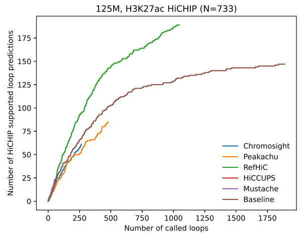

(r)

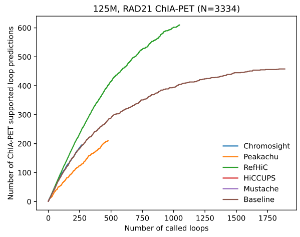

(s)

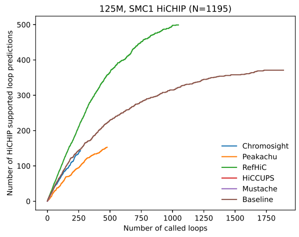

(t)

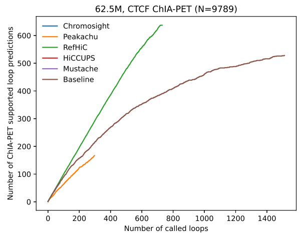

(u)

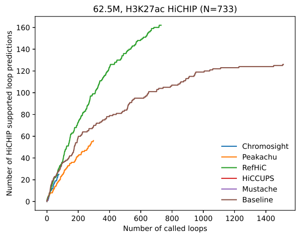

(v)

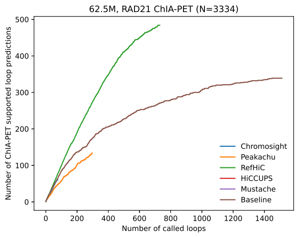

(w)

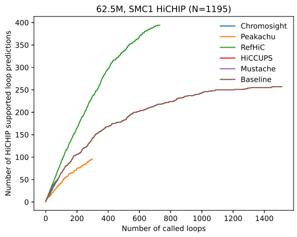

(x)

Supplementary Figure 4. Comparison of RefHiC, Chromosight, Peakachu, HiC-CUPS, Mustache, and Baseline (Supplementary Note 2) on GM12878 HiC data of lower sequencing depths. Number of ChIA-PET/HiCHIP-supported loop predictions, among the top predictions made by RefHiC and other tools, for test chromosomes chromosomes 15-17, compared against CTCF or RAD21 ChIA-PET, as well as H3K27ac or SMC1 HiCHIP. The Baseline model, which does not use a reference panel, does not perform as well as RefHiC.

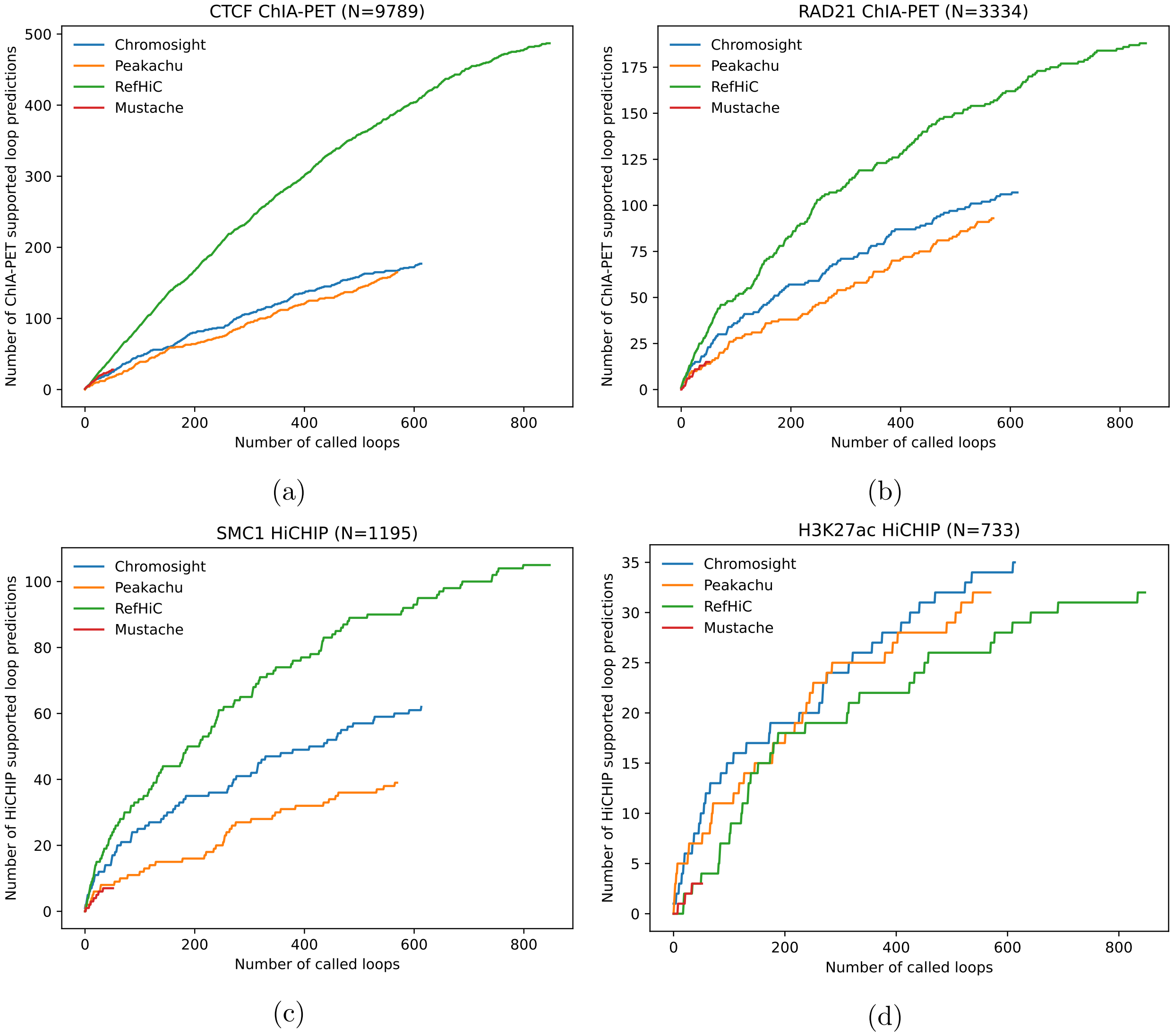

Supplementary Figure 5. Comparison of unique loops predicted by RefHiC, Chromosight, Peakachu, HiCCUPS, and Mustache on GM12878 HiC data (500M valid read pairs). Number of ChIA-PET/HiCHIP-supported loop predictions, among the top predictions made by RefHiC and other tools, for test chromosomes chr15-17, compared against CTCF ChIA-PET (a), RAD21 ChIA-PET (b), SMC1 HiCHIP (c), and H3K27ac HiCHIP (d). Only loops uniquely predicted by one of the four tools are being considered.

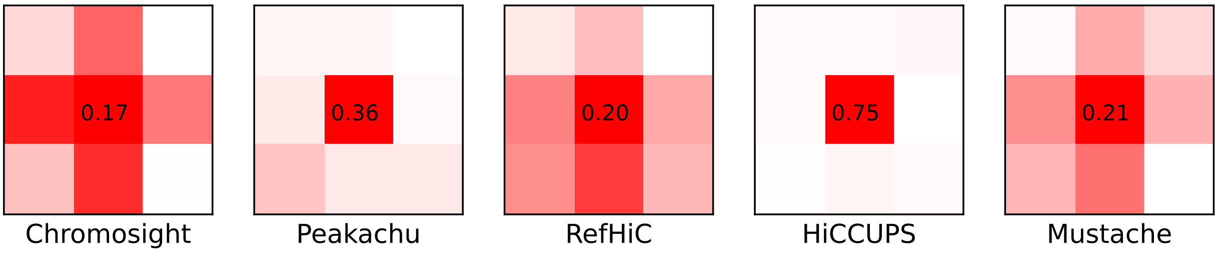

Supplementary Figure 6. For each prediction tool, the heatmap shows the fraction of predicted loops where the locally maximum interaction frequency is observed at the site of the predicted loop itself (central pixel) or at one of the 8 neighboring pixels. HiCCUPS tends to predict local maximas as loops. In contrast, only 17-36% of predicted loops produced by other tools, including RefHiC, are local maxima in terms of read counts.

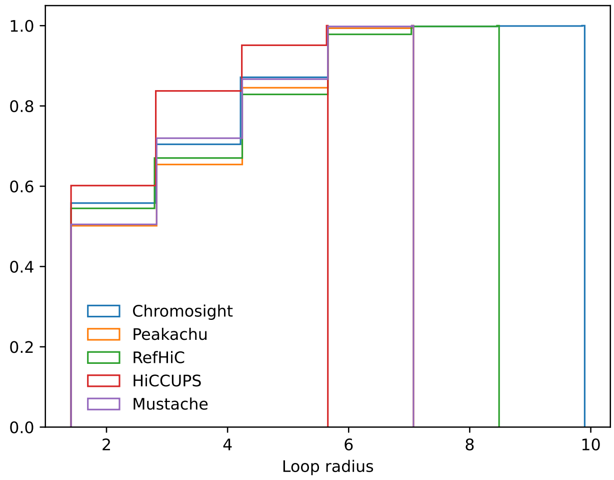

Supplementary Figure 7. Radius of loops predicted by RefHiC and other tools. The cumulative distribution of loop radius shows that HiCCUPS predicted more narrow loops than other tools, including RefHiC. In contrast, the distribution of loop sizes in Chromosight's, Peakachu's, RefHiC's, and Mustache's predictions are similar.

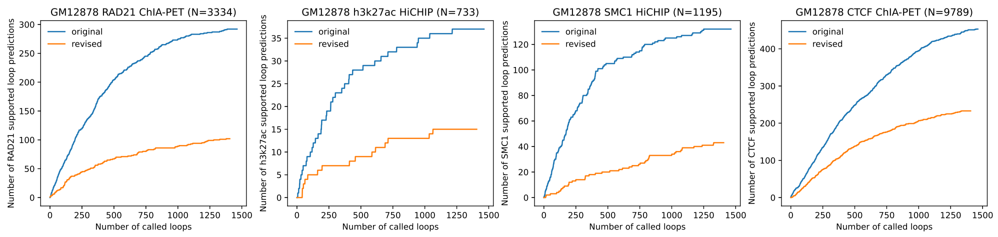

(a) RefHiC

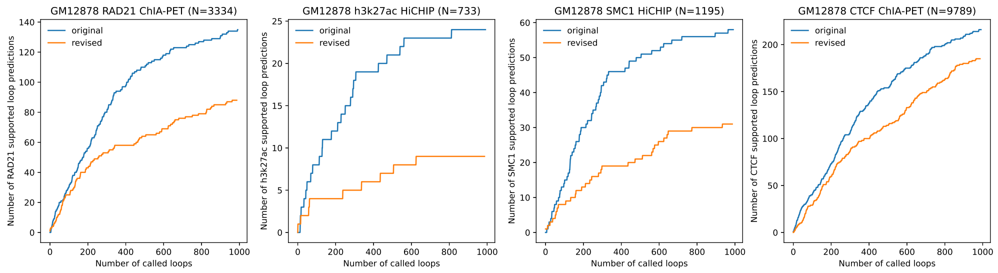

(b) Chromosight

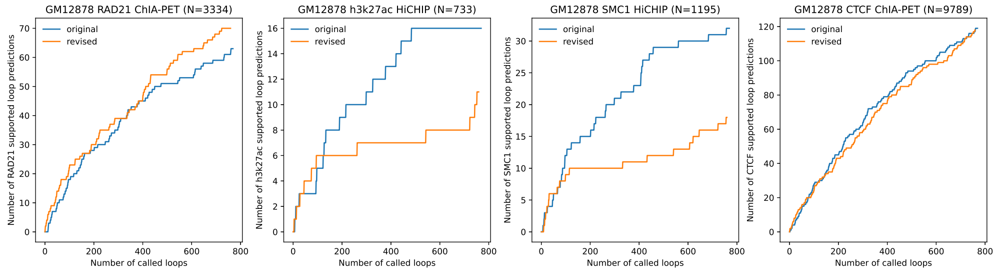

(c) Peakachu

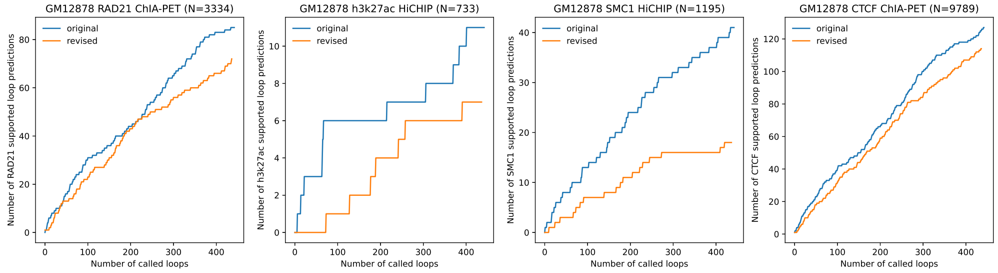

(d) Mustasche

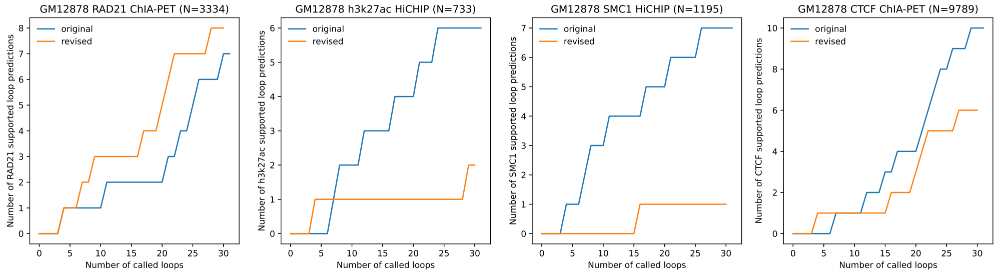

(e) HiCCUPS

Supplementary Figure 8. Comparison of the performance of different prediction tools, compared to a modified version (labeled "revised") where each loop prediction is "corrected" to the local maximum (in terms of read count) in the 3×3 sub-matrix around each original prediction.

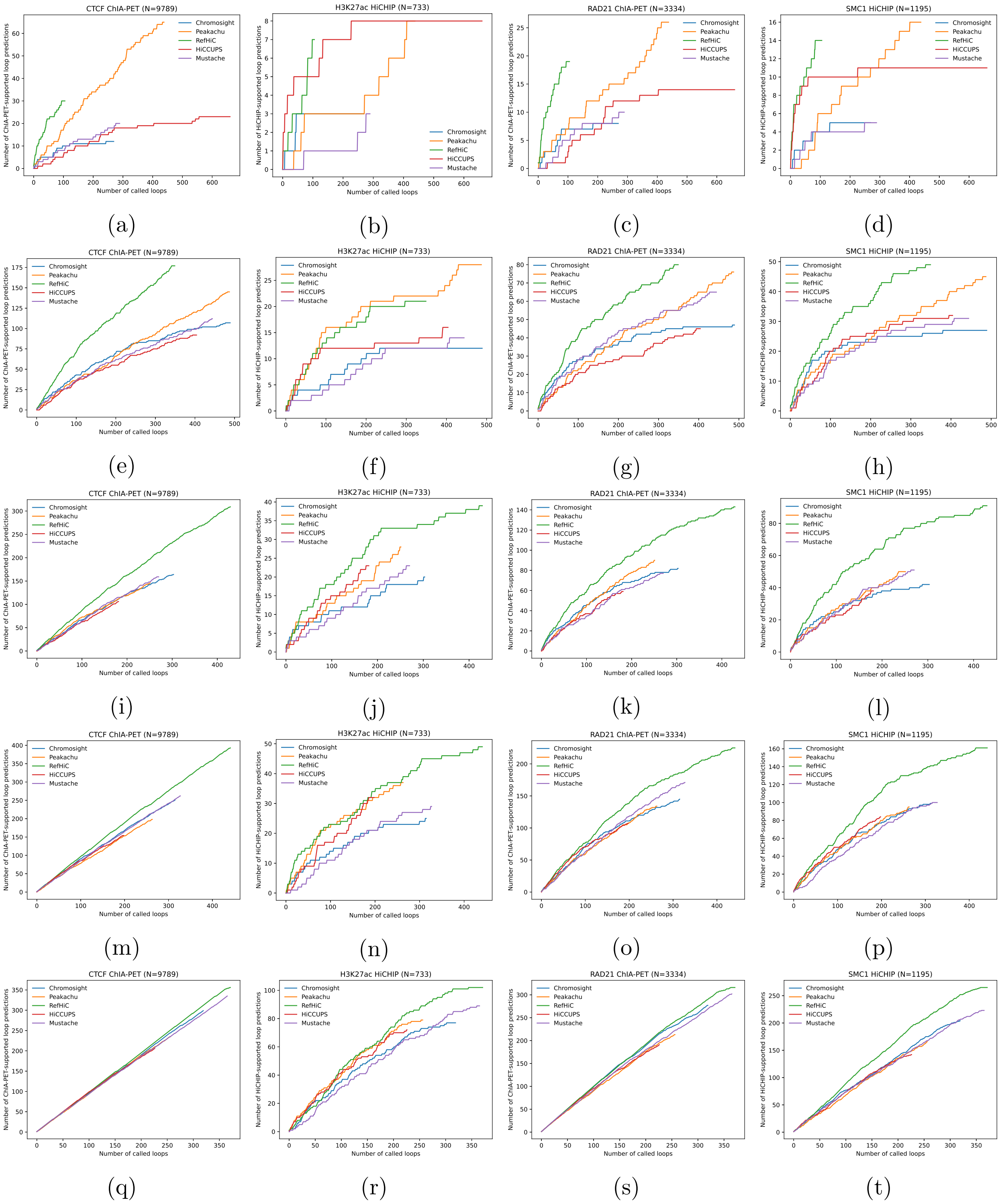

Supplementary Figure 9. Comparison of tools' ability to identify rare and common loops. Number of ChIA-PET/HiCHIP-supported loop predictions, among the top predictions of loop frequencies 0 (a-d), 1-5 (e-h), 6-10 (i-l), 11-15 (m-p), and 15-19 (q-t), made by RefHiC and other tools, for test chromosomes chr15, chr16, and chr17, compared against CTCF or RAD21 ChIA-PET, as well as H3K27ac or SMC1 HiCHIP. RefHiC's loop predictions matches those experimental data better than predictions made by other tools or equally well for both rare and common loops.

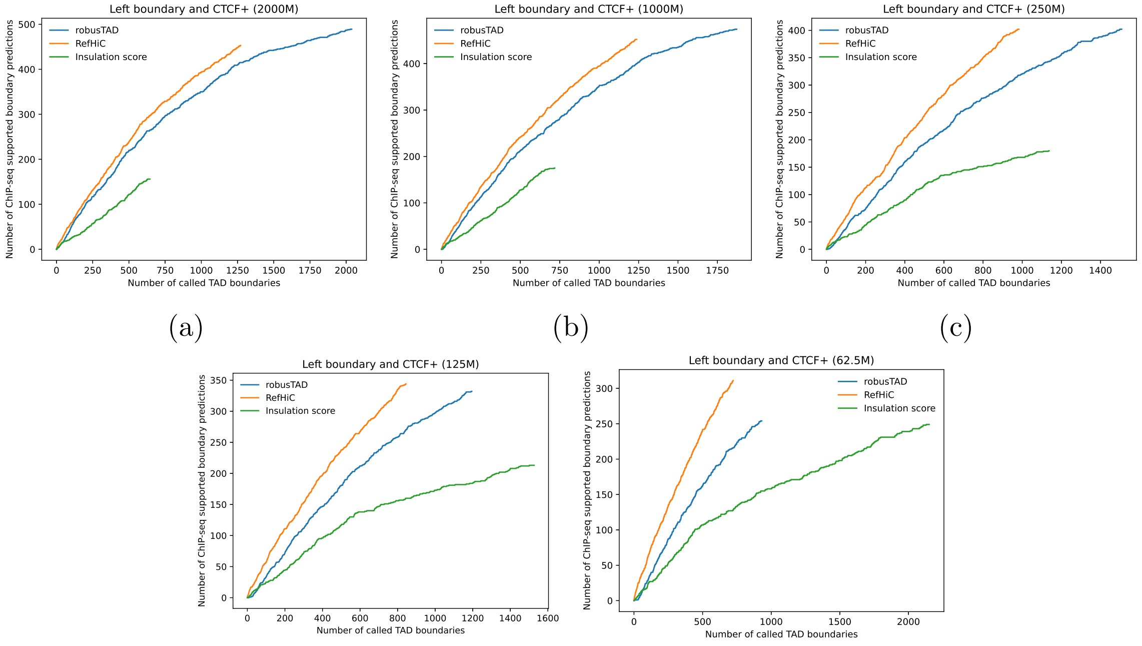

(a) (b) (c)

(d) (e)

Supplementary Figure 10. Comparison of RefHiC, robusTAD, and Insulation score on detecting left TAD boundaries from GM12878 HiC data at lower sequencing depths. Number of predicted TAD boundaries for Hi-C data containing 2,000M (a), 1,000M (b), 250M (c), 125M (d), and 62.5M (e) vaild read pairs supported by CTCF ChIP-seq data (test chromosomes chr15, chr16, and chr17).

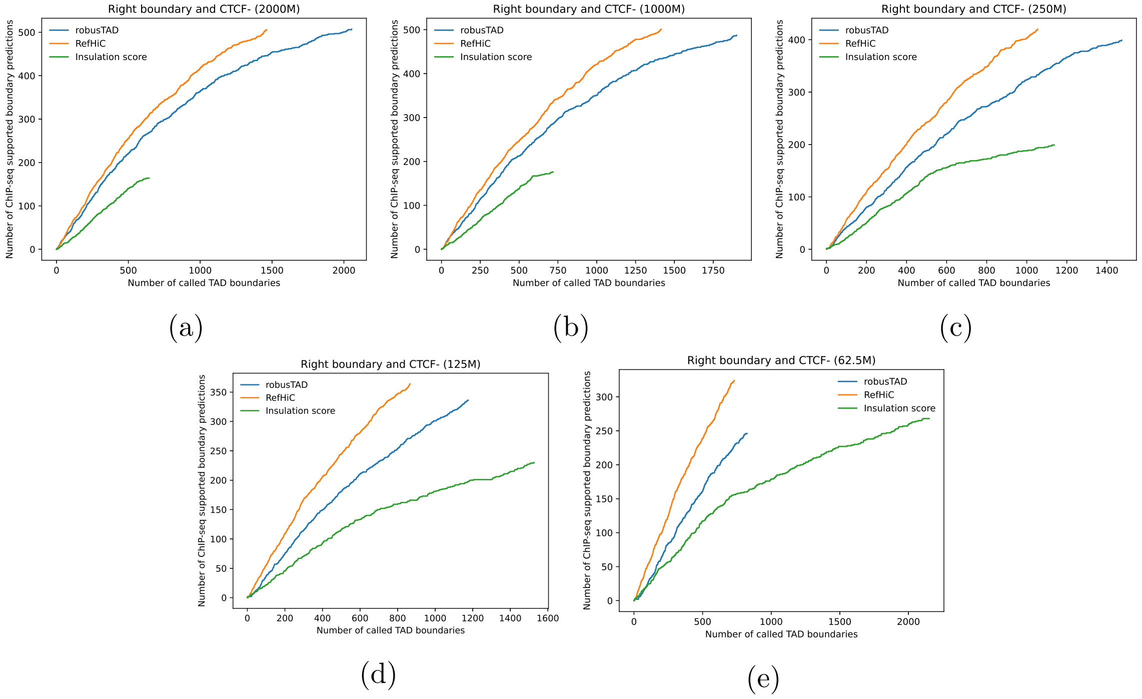

Supplementary Figure 11. Comparison of RefHiC, robusTAD, and Insulation score on detecting right TAD boundaries from GM12878 HiC data at lower sequencing depths. Number of predicted TAD boundaries for Hi-C data containing 2,000M (a), 1,000M (b), 250M (c), 125M (d), and 62.5M (e) vaild read pairs supported by CTCF ChIP-seq data (test chromosomes chr15, chr16, and chr17).

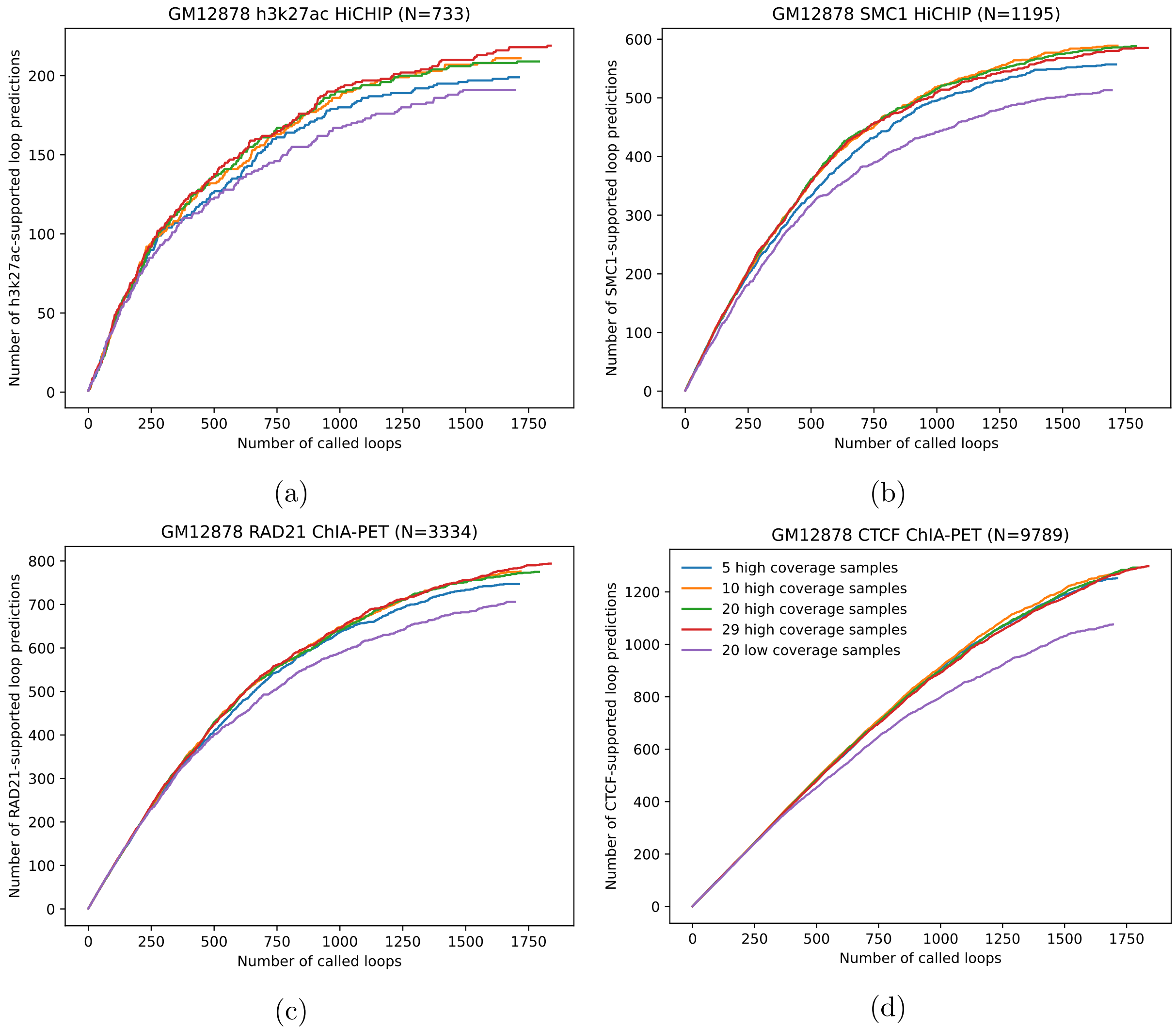

Supplementary Figure 12. RefHiC Detects loops using reference panel with different samples for GM12878 Hi-C data (500M valid read pairs). Number of ChIA-PET/HiCHIP-supported loop predictions among the top predictions made by RefHiC using reference panels containing 5, 10, 20, 20 low coverage, and 29 Hi-C samples for test chromosomes (chr15, chr16, and chr17), compared against H3K27ac HiCHIP (a), SMC1 HiCHIP (b), RAD21 ChIA-PET (c), and CTCF ChIA-PET (d) data.

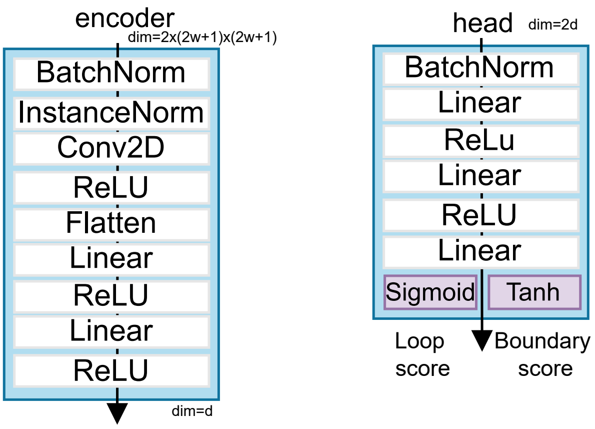

Supplementary Figure 13. Details of the encoder and head modules. The task-specific head has different output activation functions for TAD boundary (Tanh) and loop (Sigmoid). w is a hyperparameter for square size, and d is embedding dimension.

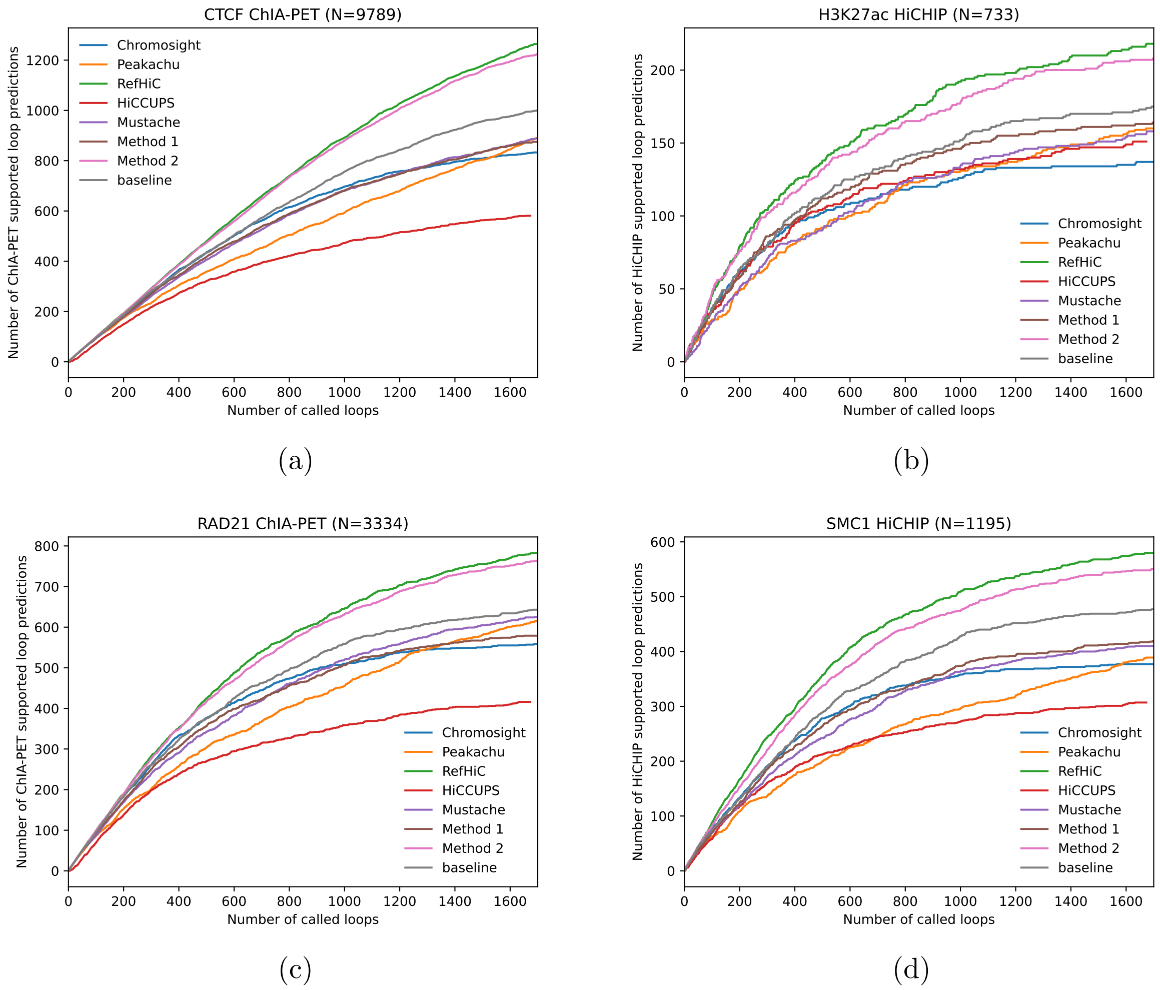

Supplementary Figure 14. Evaluating RefHiC's ability to identify loops from novel cell types by using GM12878 Hi-C data (500M valid read pairs) as input. Number of ChIA-PET/HiCHIP-supported loop predictions, among the top predictions made by RefHiC and other tools, for test chromosomes chromosomes 15-17, compared against CTCF (a) or RAD21 (c) ChIA-PET, as well as H3K27ac (b) or SMC1 (d) HiCHIP. The Baseline model is explained in Supplementary Note 2. Method 1 and 2 as explained in Supplementary Note 4 are used to evaluate RefHiC's ability to identify loops from novel cell types.

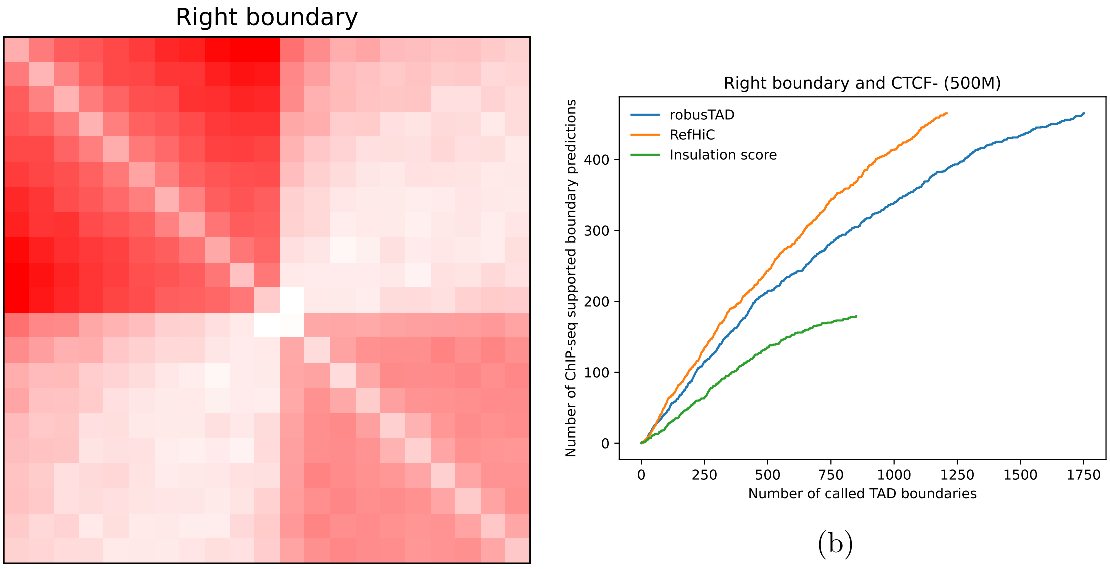

(a)

(b)

Supplementary Figure 15. Detection of TAD boundaries on GM12878 Hi-C data (500 valid read pairs). a, TAD boundary pileups for right (c) boundaries predicted by RefHiC. **b** Number of RefHiC, RobusTAD, and Insulation score predicted right TAD boundaries supported by ChIP-seq identified CTCF binding sites on the reverse (CTCF-) strand.

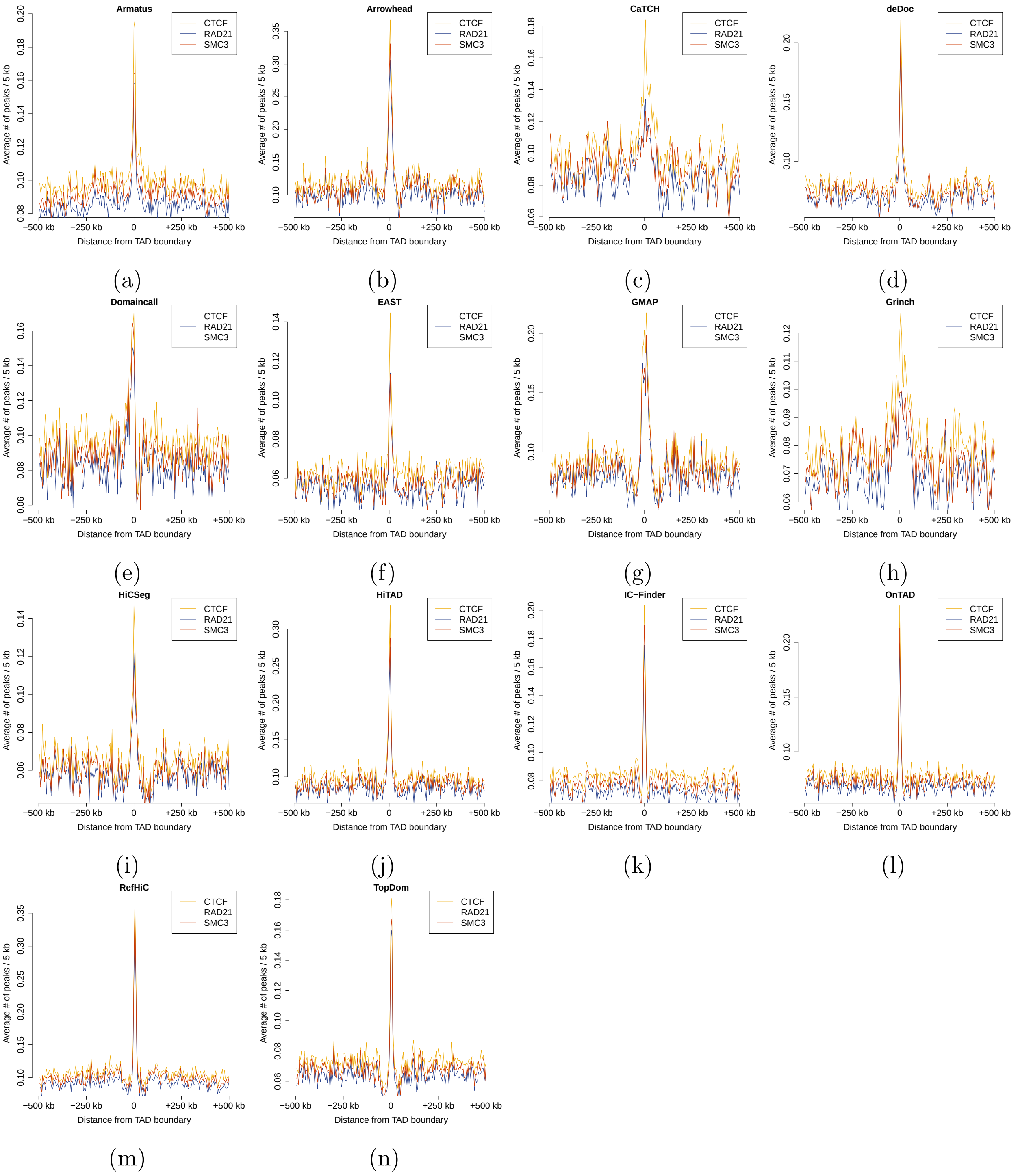

Supplementary Figure 16. ChIP-seq peak signals for CTCF, RAD21, and SMC3 around TAD boundaries annotated by each tool.

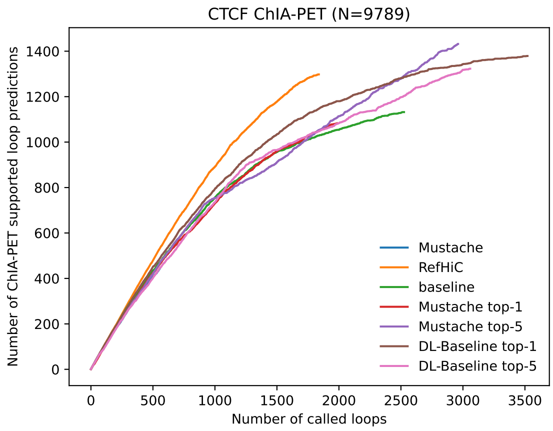

(a)

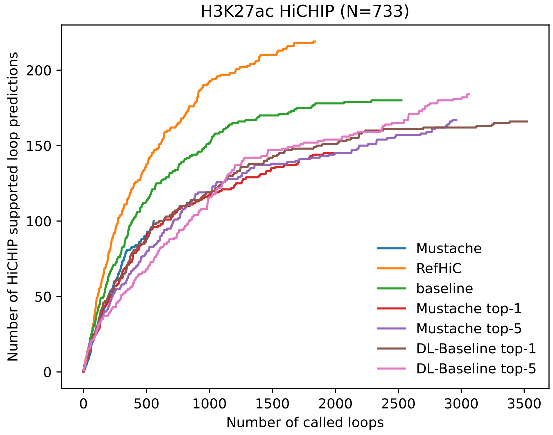

(b)

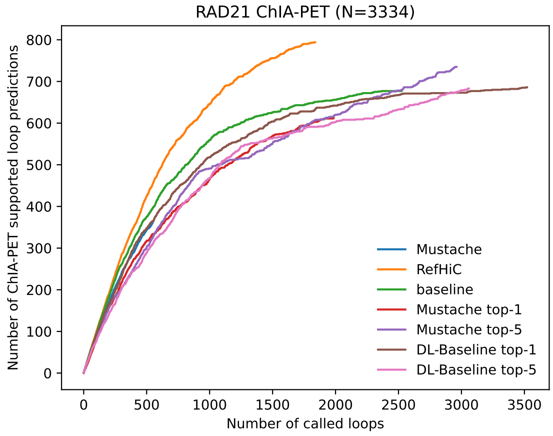

(c)

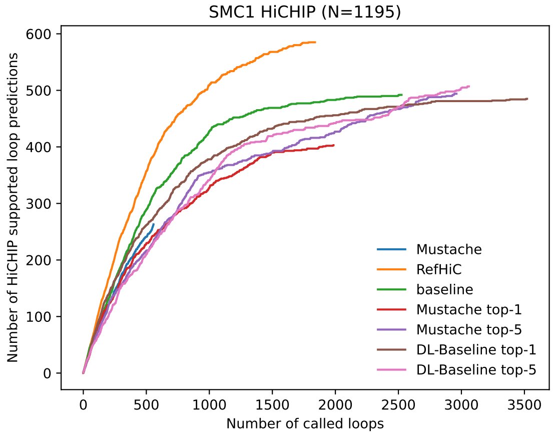

(d)

Supplementary Figure 17. Comparison of loops predicted by RefHiC, baseline, Mustache, and four reference only approaches described in Supplementary Note 5 on GM12878 HiC data (500M valid read pairs). Number of ChIA-PET/HiCHIP-supported loop predictions, among predictions made by each tool for test chromosomes 15-17 compared against CTCF ChIA-PET (a), H3K27ac HiCHIP (b), RAD21 ChIA-PET (c), and SMC1 HiCHIP (d). RefHiC's loop predictions matches all data better than predictions made by alternative methods.

References

[1] S. L. Battle, N. Doni Jayavelu, R. N. Azad, J. Hesson, F. N. Ahmed, E. G. Overbey, J. A. Zoller, J. Mathieu, H. Ruohola-Baker, C. B. Ware, and R. D. Hawkins. Enhancer Chromatin and 3D Genome Architecture Changes from Naive to Primed Human Embryonic Stem Cell States. *Stem Cell Reports*, 12(5):1129–1144, 05 2019.

[2] Boyan Bonev, Netta Mendelson Cohen, Quentin Szabo, Lauriane Fritsch, Giorgio L Papadopoulos, Yaniv Lubling, Xiaole Xu, Xiaodan Lv, Jean-Philippe Hugnot, Amos Tanay, et al. Multiscale 3d genome rewiring during mouse neural development. *Cell*, 171(3):557–572, 2017.

[3] K. L. Bunting, T. D. Soong, R. Singh, Y. Jiang, W. Béguelin, D. W. Poloway, B. L. Swed, K. Hatzi, W. Reisacher, M. Teater, O. Elemento, and A. M. Melnick. Multi-tiered Reorganization of the Genome during B Cell Affinity Maturation Anchored by a Germinal Center-Specific Locus Control Region. *Immunity*, 45(3):497–512, 09 2016.

[4] Christopher JF Cameron, Josee Dostie, and Mathieu Blanchette. Estimating dna-dna interaction frequency from hi-c data at restriction-fragment resolution. *bioRxiv*, page 377523, 2018.

[5] ENCODE Project Consortium et al. An integrated encyclopedia of dna elements in the human genome. *Nature*, 489(7414):57, 2012.

[6] J. R. Dixon, I. Jung, S. Selvaraj, Y. Shen, J. E. Antosiewicz-Bourget, A. Y. Lee, Z. Ye, A. Kim, N. Rajagopal, W. Xie, Y. Diao, J. Liang, H. Zhao, V. V. Lobanenkov, J. R. Ecker, J. A. Thomson, and B. Ren. Chromatin architecture reorganization during stem cell differentiation. *Nature*, 518(7539):331–336, Feb 2015.

[7] Y. Guo, A. A. Perez, D. J. Hazelett, G. A. Coetzee, S. K. Rhie, and P. J. Farnham. CRISPR-mediated deletion of prostate cancer risk-associated CTCF loop anchors identifies repressive chromatin loops. *Genome Biol*, 19(1):160, 10 2018.

[8] J. H. I. Haarhuis, R. H. van der Weide, V. A. Blomen, J. O. Yáñez-Cuna, M. Amendola, M. S. van Ruiten, P. H. L. Krijger, H. Teunissen, R. H. Medema, B. van Steensel, T. R. Brummelkamp, E. de Wit, and B. D. Rowland. The Cohesin Release Factor WAPL Restricts Chromatin Loop Extension. *Cell*, 169(4):693–707, 05 2017.

[9] S. Heinz, L. Texari, M. G. B. Hayes, M. Urbanowski, M. W. Chang, N. Givarkes, A. Rialdi, K. M. White, R. A. Albrecht, L. Pache, I. Marazzi, A. García-Sastre, M. L. Shaw, and C. Benner. Transcription Elongation Can Affect Genome 3D Structure. *Cell*, 174(6):1522–1536, 09 2018.

[10] Hui Huang, Quan Zhu, Adam Jussila, Yuanyuan Han, Bogdan Bintu, Colin Kern, Mattia Conte, Yanxiao Zhang, Simona Bianco, Andrea M Chiariello, et al. Ctcf mediates dosage-and sequence-context-dependent transcriptional insulation by forming local chromatin domains. *Nature genetics*, 53(7):1064–1074, 2021.

[11] E. C. Jacobson, J. K. Perry, D. S. Long, A. L. Olins, D. E. Olins, B. E. Wright, M. H. Vickers, and J. M. O'Sullivan. Migration through a small pore disrupts inactive chromatin organization in neutrophil-like cells. *BMC Biol*, 16(1):142, 11 2018.

[12] I. Jung, A. Schmitt, Y. Diao, A. J. Lee, T. Liu, D. Yang, C. Tan, J. Eom, M. Chan, S. Chee, Z. Chiang, C. Kim, E. Masliah, C. L. Barr, B. Li, S. Kuan, D. Kim, and B. Ren. A compendium of promoter-centered long-range chromatin interactions in the human genome. *Nat Genet*, 51(10):1442–1449, 10 2019.

[13] Naoki Kubo, Haruhiko Ishii, Xiong Xiong, Simona Bianco, Franz Meitinger, Rong Hu, James D Hocker, Mattia Conte, David Gorkin, Miao Yu, et al. Promoter-proximal ctcf binding promotes distal enhancer-dependent gene activation. *Nature structural & molecular biology*, 28(2):152–161, 2021.

[14] S. Lalonde, V. A. Codina-Fauteux, S. M. de Bellefon, F. Leblanc, M. Beaudoin, M. M. Simon, R. Dali, T. Kwan, K. S. Lo, T. Pastinen, and G. Lettre. Integrative analysis of vascular endothelial cell genomic features identifies AIDA as a coronary artery disease candidate gene. *Genome Biol*, 20(1):133, 07 2019.

[15] D. Leung, I. Jung, N. Rajagopal, A. Schmitt, S. Selvaraj, A. Y. Lee, C. A. Yen, S. Lin, Y. Lin, Y. Qiu, W. Xie, F. Yue, M. Hariharan, P. Ray, S. Kuan, L. Edsall, H. Yang, N. C. Chi, M. Q. Zhang, J. R. Ecker, and B. Ren. Integrative analysis of haplotype-resolved epigenomes across human tissues. *Nature*, 518(7539):350–354, Feb 2015.

[16] Dejun Lin, Justin Sanders, and William Stafford Noble. Hicrep. py: fast comparison of hi-c contact matrices in python. *Bioinformatics*, 37(18):2996–2997, 2021.

[17] Robin E Lindeman, Mark W Murphy, Kellie S Agrimson, Rachel L Gewiss, Vivian J Bardwell, Micah D Gearhart, and David Zarkower. The conserved sex regulator dmrt1 recruits sox9 in sexual cell fate reprogramming. *Nucleic acids research*, 49(11):6144–6164, 2021.

[18] Qiao Liu, Hairong Lv, and Rui Jiang. hicgan infers super resolution hi-c data with generative adversarial networks. *Bioinformatics*, 35(14):i99–i107, 2019.

[19] Tong Liu and Zheng Wang. Hicnn: a very deep convolutional neural network to better enhance the resolution of hi-c data. *Bioinformatics*, 2019.

[20] Kevin Monahan, Adan Horta, and Stavros Lomvardas. Lhx2-and ldb1-mediated trans interactions regulate olfactory receptor choice. *Nature*, 565(7740):448–453, 2019.

[21] Maxwell R Mumbach, Adam J Rubin, Ryan A Flynn, Chao Dai, Paul A Khavari, William J Greenleaf, and Howard Y Chang. Hichip: efficient and sensitive analysis of protein-directed genome architecture. *Nature methods*, 13(11):919–922, 2016.

[22] Maxwell R Mumbach, Ansuman T Satpathy, Evan A Boyle, Chao Dai, Benjamin G Gowen, Seung Woo Cho, Michelle L Nguyen, Adam J Rubin, Jeffrey M Granja, Katelynn R Kazane, et al. Enhancer connectome in primary human cells identifies target genes of disease-associated dna elements. *Nature genetics*, 49(11):1602–1612, 2017.

[23] G. Nir, I. Farabella, C. Pérez Estrada, C. G. Ebeling, B. J. Beliveau, H. M. Sasaki, S. D. Lee, S. C. Nguyen, R. B. McCole, S. Chattoraj, J. Erceg, J. AlHaj Abed, N. M. C. Martins, H. Q. Nguyen, M. A. Hannan, S. Russell, N. C. Durand, S. S. P. Rao, J. Y. Kishi, P. Soler-Vila, M. Di Pierro, J. N. Onuchic, S. P. Callahan, J. M. Schreiner, J. A. Stuckey, P. Yin, E. L. Aiden, M. A. Marti-Renom, and C. T. Wu. Walking along chromosomes with super-resolution imaging, contact maps, and integrative modeling. *PLoS Genet*, 14(12):e1007872, 12 2018.

[24] S. S. Rao, M. H. Huntley, N. C. Durand, E. K. Stamenova, I. D. Bochkov, J. T. Robinson, A. L. Sanborn, I. Machol, A. D. Omer, E. S. Lander, and E. L. Aiden. A 3D map of the human genome at kilobase resolution reveals principles of chromatin looping. *Cell*, 159(7):1665–1680, Dec 2014.

[25] S. S. P. Rao, S. C. Huang, B. Glenn St Hilaire, J. M. Engreitz, E. M. Perez, K. R. Kieffer-Kwon, A. L. Sanborn, S. E. Johnstone, G. D. Bascom, I. D. Bochkov, X. Huang, M. S. Shamim, J. Shin, D. Turner, Z. Ye, A. D. Omer, J. T. Robinson, T. Schlick, B. E. Bernstein, R. Casellas, E. S. Lander, and E. L. Aiden. Cohesin Loss Eliminates All Loop Domains. *Cell*, 171(2):305–320, Oct 2017.

[26] Suhas SP Rao, Miriam H Huntley, Neva C Durand, Elena K Stamenova, Ivan D Bochkov, James T Robinson, Adrian L Sanborn, Ido Machol, Arina D Omer, Eric S Lander, et al. A 3d map of the human genome at kilobase resolution reveals principles of chromatin looping. *Cell*, 159(7):1665–1680, 2014.

[27] Abbas Roayaei Ardakany, Halil Tuvan Gezer, Stefano Lonardi, and Ferhat Ay. Mustache: multi-scale detection of chromatin loops from hi-c and micro-c maps using scale-space representation. *Genome biology*, 21(1):1–17, 2020.

[28] C. D. Rosencrance, H. N. Ammouri, Q. Yu, T. Ge, E. J. Rendleman, S. A. Marshall, and K. P. Eagen. Chromatin Hyperacetylation Impacts Chromosome Folding by Forming a Nuclear Subcompartment. *Mol Cell*, 78(1):112–126, 04 2020.

[29] S. Sati, B. Bonev, Q. Szabo, D. Jost, P. Bensadoun, F. Serra, V. Loubiere, G. L. Papadopoulos, J. C. Rivera-Mulia, L. Fritsch, P. Bouret, D. Castillo, J. L. Gelpi, M. Orozco, C. Vaillant, F. Pellestor, F. Bantignies, M. A. Marti-Renom, D. M. Gilbert, J. M. Lemaitre, and G. Cavalli. 4D Genome Rewiring during Oncogene-Induced and Replicative Senescence. *Mol Cell*, 78(3):522–538, 05 2020.

[30] G. Stik, E. Vidal, M. Barrero, S. Cuartero, M. Vila-Casadesús, J. Mendieta-Esteban, T. V. Tian, J. Choi, C. Berenguer, A. Abad, B. Borsari, F. le Dily, P. Cramer, M. A. Marti-Renom, R. Stadhouders, and T. Graf. CTCF is dispensable for immune cell transdifferentiation but facilitates an acute inflammatory response. *Nat Genet*, 52(7):655–661, 07 2020.

[31] Zhonghui Tang, Oscar Junhong Luo, Xingwang Li, Meizhen Zheng, Jacqueline Jufen Zhu, Przemyslaw Szalaj, Pawel Trzaskoma, Adriana Magalska, Jakub Wlodarczyk, Blazej Ruszczycki, et al. Ctcf-mediated human 3d genome architecture reveals chromatin topology for transcription. *Cell*, 163(7):1611–1627, 2015.

[32] L. Tian, Y. Shao, S. Nance, J. Dang, B. Xu, X. Ma, Y. Li, B. Ju, L. Dong, S. Newman, X. Zhou, P. Schreiner, E. Tseng, T. Hon, M. Ashby, C. Li, J. Easton, T. A. Gruber, and J. Zhang. Long-read sequencing unveils IGH-DUX4 translocation into the silenced IGH allele in B-cell acute lymphoblastic leukemia. *Nat Commun*, 10(1):2789, 06 2019.

[33] Stéfan van der Walt, Johannes L. Schönberger, Juan Nunez-Iglesias, François Boulogne, Joshua D. Warner, Neil Yager, Emmanuelle Gouillart, Tony Yu, and the scikit-image contributors. scikit-image: image processing in Python. *PeerJ*, 2:e453, 6 2014.

[34] Laura Vian, Aleksandra Pekowska, Suhas SP Rao, Kyong-Rim Kieffer-Kwon, Seolkyoung Jung, Laura Baranello, Su-Chen Huang, Laila El Khattabi, Marei Dose, Nathanael Pruett, et al. The energetics and physiological impact of cohesin extrusion. *Cell*, 173(5):1165–1178, 2018.

[35] G. Wutz, R. Ladurner, B. G. St Hilaire, R. R. Stocsits, K. Nagasaka, B. Pignard, A. Sanborn, W. Tang, C. Várnai, M. P. Ivanov, S. Schoenfelder, P. van der Lelij, X. Huang, G. Dürnberger, E. Roitinger, K. Mechtler, I. F. Davidson, P. Fraser, E. Lieberman-Aiden, and J. M. Peters. from WAPL. *Elife*, 9, 02 2020.

[36] Jian Yan, Shi-An A Chen, Andrea Local, Tristin Liu, Yunjiang Qiu, Kristel M Dorighi, Sebastian Preissl, Chloe M Rivera, Chaochen Wang, Zhen Ye, et al. Histone h3 lysine 4 monomethylation modulates long-range chromatin interactions at enhancers. *Cell research*, 28(2):204–220, 2018.

[37] Tao Yang, Feipeng Zhang, Galip Gürkan Yardımcı, Fan Song, Ross C Hardison, William Stafford Noble, Feng Yue, and Qunhua Li. Hicrep: assessing the reproducibility of hi-c data using a stratum-adjusted correlation coefficient. *Genome research*, 27(11):1939–1949, 2017.

[38] Shanshan Zhang, Dylan Plummer, Leina Lu, Jian Cui, Wanying Xu, Miao Wang, Xiaoxiao Liu, Nachiketh Prabhakar, Jatin Shrinet, Divyaa Srinivasan, et al. Deeploop robustly maps chromatin interactions from sparse allele-resolved or single-cell hi-c data at kilobase resolution. *Nature Genetics*, pages 1–13, 2022.

[39] Y. Zhang, T. Li, S. Preissl, M. L. Amaral, J. D. Grinstein, E. N. Farah, E. Destici, Y. Qiu, R. Hu, A. Y. Lee, S. Chee, K. Ma, Z. Ye, Q. Zhu, H. Huang, R. Fang, L. Yu, J. C. Izpisua Belmonte, J. Wu, S. M. Evans, N. C. Chi, and B. Ren. Transcriptionally active HERV-H retrotransposons demarcate topologically associating domains in human pluripotent stem cells. *Nat Genet*, 51(9):1380–1388, 09 2019.

[40] Yan Zhang, Lin An, Jie Xu, Bo Zhang, W Jim Zheng, Ming Hu, Jijun Tang, and Feng Yue. Enhancing hi-c data resolution with deep convolutional neural network hicplus. *Nature communications*, 9(1):750, 2018.
