# Supplementary material for: Reference panel guided topological structure annotation of Hi-C data
Source: Nat Commun. 2022 Dec 2;13:7426. doi: 10.1038/s41467-022-35231-3 (PMC9718747; doi:10.1038/s41467-022-35231-3)
Supplement: Supplementary file 3 — Reporting Summary [file 41467_2022_35231_MOESM3_ESM.pdf]

nature portfolio

Corresponding author(s): Mathieu Blanchette

Last updated by author(s): Nov 10, 2022

Reporting Summary

Nature Portfolio wishes to improve the reproducibility of the work that we publish. This form provides structure for consistency and transparency in reporting. For further information on Nature Portfolio policies, see our Editorial Policies and the Editorial Policy Checklist.

Statistics

For all statistical analyses, confirm that the following items are present in the figure legend, table legend, main text, or Methods section.

n/a	Confirmed	
☒	☐	The exact sample size (*n*) for each experimental group/condition, given as a discrete number and unit of measurement
☐	☒	A statement on whether measurements were taken from distinct samples or whether the same sample was measured repeatedly
☐	☒	The statistical test(s) used AND whether they are one- or two-sided<br>*Only common tests should be described solely by name; describe more complex techniques in the Methods section.*
☒	☐	A description of all covariates tested
☒	☐	A description of any assumptions or corrections, such as tests of normality and adjustment for multiple comparisons
☒	☐	A full description of the statistical parameters including central tendency (e.g. means) or other basic estimates (e.g. regression coefficient) AND variation (e.g. standard deviation) or associated estimates of uncertainty (e.g. confidence intervals)
☐	☒	For null hypothesis testing, the test statistic (e.g. *F*, *t*, *r*) with confidence intervals, effect sizes, degrees of freedom and *P* value noted<br>*Give P values as exact values whenever suitable.*
☒	☐	For Bayesian analysis, information on the choice of priors and Markov chain Monte Carlo settings
☒	☐	For hierarchical and complex designs, identification of the appropriate level for tests and full reporting of outcomes
☒	☐	Estimates of effect sizes (e.g. Cohen's *d*, Pearson's *r*), indicating how they were calculated

Our web collection on statistics for biologists contains articles on many of the points above.

Software and code

Policy information about availability of computer code

Data collection	We downloaded ChIP-Seq data from ENCODE (https://www.encodeproject.org/), UCSC Genome Browser (https://genome.ucsc.edu/), and 4DN Data Portal (https://data.4dnucleome.org/) with wget and curl.
Data analysis	The software in this manuscript is publicly available at https://github.com/BlanchetteLab/RefHiC, and instructions for reproducing all the results in the manuscript are at https://zenodo.org/record/7133194. It relies on cooler (0.8.11), PyTorch (1.11.0), NumPy (1.19.5), matplotlib (3.3.4), einops (0.3.2),appdirs (1.4.4), click (8.0.1), pandas (1.3.5)

For manuscripts utilizing custom algorithms or software that are central to the research but not yet described in published literature, software must be made available to editors and reviewers. We strongly encourage code deposition in a community repository (e.g. GitHub). See the Nature Portfolio guidelines for submitting code & software for further information.

Data

Policy information about availability of data

All manuscripts must include a data availability statement. This statement should provide the following information, where applicable:

- Accession codes, unique identifiers, or web links for publicly available datasets
- A description of any restrictions on data availability
- For clinical datasets or third party data, please ensure that the statement adheres to our policy

All data analyzed during this study are included in this published article (and its supplementary information files): All data included in the reference panels of this study are summarized in Supplementary Tables 1, 2, and 3 with references and/or accession codes. They are all publicly available at GEO, ENCODE or 4DN data

repository, or at http://repo.cs.mcgill.ca/PUB/blanchem/RefHiC. We trained and evaluated RefHiC using public available contact maps including GM12878 (4DNFIXP4QG5B), K562 (4DNFI4DGNY7J), IMR-90 (4DNFIJTOIGOI), HCT-116 (4DNFILP99QJS), mESC (4DNFIDA2WGV8).

Human research participants

Policy information about studies involving human research participants and Sex and Gender in Research.

Reporting on sex and gender	This study does not involve human research participants.
Population characteristics	This study does not involve human research participants.
Recruitment	This study does not involve human research participants.
Ethics oversight	This study does not involve human research participants.

Note that full information on the approval of the study protocol must also be provided in the manuscript.

Field-specific reporting

Please select the one below that is the best fit for your research. If you are not sure, read the appropriate sections before making your selection.

[x] Life sciences [ ] Behavioural & social sciences [ ] Ecological, evolutionary & environmental sciences

For a reference copy of the document with all sections, see nature.com/documents/nr-reporting-summary-flat.pdf

Life sciences study design

All studies must disclose on these points even when the disclosure is negative.

Sample size	We do not need to determine the sample size in this study.
Data exclusions	No data were excluded from the analysis.
Replication	Our software is open source and was tested on major Unix-like operating systems. We performed TAD and loop detections from different Hi-C data of different sequence depths and confirmed the reproducibility and provided results in Figure 4, Suppl. Figures 4, 10, and 11.
Randomization	This is not relevant to our study. Our study does not involve any experimental group allocations.
Blinding	This is not relevant to our study. Our study does not involve any group allocations.

Reporting for specific materials, systems and methods

We require information from authors about some types of materials, experimental systems and methods used in many studies. Here, indicate whether each material, system or method listed is relevant to your study. If you are not sure if a list item applies to your research, read the appropriate section before selecting a response.

Materials & experimental systems

n/a	Involved in the study	
[x]	[ ]	Antibodies
[x]	[ ]	Eukaryotic cell lines
[x]	[ ]	Palaeontology and archaeology
[x]	[ ]	Animals and other organisms
[x]	[ ]	Clinical data
[x]	[ ]	Dual use research of concern

Methods

n/a	Involved in the study	
[x]	[ ]	ChIP-seq
[x]	[ ]	Flow cytometry
[x]	[ ]	MRI-based neuroimaging
